# The implementation of community-based diabetes and hypertension management care program in Indonesia

**DOI:** 10.1371/journal.pone.0227806

**Published:** 2020-01-14

**Authors:** Levina Chandra Khoe, Grace Wangge, Pradana Soewondo, Dicky L. Tahapary, Indah Suci Widyahening

**Affiliations:** 1 Community Medicine Department, Faculty of Medicine, Universitas Indonesia, Jakarta, Indonesia; 2 Southeast Asian Ministers of Education Organization – Regional Centre for Food and Nutrition (SEAMEO-RECFON)/Pusat Kajian Gizi Regional (PKGR), Universitas Indonesia, Jakarta, Indonesia; 3 Division of Metabolic Endocrine, Department of Internal Medicine, Facuty of Medicine, Universitas Indonesia, Jakarta, Indonesia; Institute of Economic Growth, INDIA

## Abstract

Since 2010, Indonesian government has initiated a chronic disease management program, Prolanis (*Program Pengendalian Penyakit Kronis*) targeted for diabetes and hypertension. The program is continued at the commencement of universal health coverage (UHC) in 2014. “This study aimed to report the utilization and cost of the implementation of Prolanis in Indonesia from 2014 to 2016, or two years since the commencement of Indonesian universal health coverage.” Secondary data analysis was performed using publicly available data and data obtained from the national health insurance agency (BPJS); while data on disease prevalence were collected from basic national health survey. There was an increase trend of Prolanis participants, from around 11,000 participants in 2014 to more than 250,000 in 2016. More than 70% of participants were adults living in Java, however, the acceptance rate was very low in other area. Across different activities in Prolanis, physical activity was the most participated ones. In comparison to other regions, regions in Java were the most active area. The total expenditure for Prolanis program in 2016 increased almost triple from the annual cost in 2014. However, the cost per person was actually decreased more than 50%. Within two years of UHC implementation, there were increase covered participants and total costs, but cost per individual was decreased and there was significant difference in of cost between Java and outside Java. Further study and routine monitoring-evaluation process by health authority is needed to assess whether the cost difference would affect the service quality.

## Background

Chronic diseases prevalence has been rising rapidly, especially in middle and low-middle income countries. Four of the most prominent chronic diseases–cardiovascular diseases (CVD), cancer, chronic obstructive pulmonary disease and diabetes mellitus–are linked by common and preventable physiological risk factors, notably high blood pressure, high blood cholesterol and overweight, and by related major behavioral risk factors such as unhealthy diet, physical inactivity and tobacco use. Efforts to reduce these chronic diseases should focus on controlling the key risk factors in a well-integrated manner. [1]

Since 2010, Indonesian government has initiated a chronic disease management program (*Program Pengendalian Penyakit Kronis*-Prolanis) targeted for patients with diabetes and hypertension patients, under the civil service health insurance scheme. The program aims to prevent chronic disease patients fall into severe complications. Hence, the program was still limited to civil servants. With the commencement of universal health coverage in 2014, Prolanis has since been implemented to all patients under the national health insurance scheme. Patients with chronic diseases are able to register to this program and receive the following services: 1) medical consultation/health education; 2) regular health status monitoring; 3) home visit; 4) reminder through mobile short message service (SMS) gateway; 5) club activity; and 6) monthly routine drugs.[2] This chronic disease management program is a multi-faceted interventions that involve both, pharmacologic and non-pharmacologic strategies (e.g. patient education, physical activity, monitoring, and reminder system). The program is held by primary care centers, which already in collaboration with the national health insurance agency. The primary care centers include community health care centers (*Puskesmas*), primary care clinics, and private doctors.

Previously, some evaluation of Prolanis in Palembang showed that implementation of the program is still suboptimal.[3] One of the issues highlighted in the study was the inability to organize activity clubs due to lack of manpower and facilities. Furthermore, despite of the program, based on the national health survey data, there was an increase of diabetes prevalence diagnosed by medical doctors from 1.5 in 2013 to 2.0 in 2018. [4]This study aimed to report the utilization and cost of Prolanis in Indonesia from 2014 to 2016, or three years since the commencement of Indonesian universal health coverage.

## Methods

This is a cross-sectional study using secondary data analysis using data on number of Prolanis participants, activities and budget obtained from the national health insurance agency (BPJS) from 2014–2016. To obtain the data we had submitted a formal request to the agency.

Prolanis participants were classified based on their registered healthcare facilities, i.e. primary healthcare center (PHC), private practice, and primary care clinic. Areas in Indonesia were classified into 13 regions, i.e. 1) Aceh and North Sumatra; 2) Jambi, Riau, West Sumatra; 3) Bengkulu, Bangka Belitung, South Sumatra; 4) Banten, Jakarta (and its satellite cities; 5) West Java; 6) Jogjakarta, Central Java; 7) East Java; 8) South Kalimantan, Central Kalimantan, East Kalimantan, and North Kalimantan; 9) Maluku, West Sulawesi, South Sulawesi, Southeast Sulawesi; 10) Gorontalo, North Maluku, Central Sulawesi, North Sulawesi; 11) Bali, West Nusa Tenggara, East Nusa Tenggara; 12) Papua, West Papua; and 13) Banten, West Kalimantan, Lampung. The Regio 13 was newly created in 2015. We collected data on Prolanis activity in each region. Information on costs incurred in Prolanis was collected from BPJS records in the period of 2014–2016.

We look at the difference of Prolanis participants, activities and budget spent between regions that are located in Java Island and Outside Java. Java Island is the most populated and developed island in Indonesia, thus have a better access to health care. We think it would be interesting to see whether differences in Prolanis activities existed between regions located in Java and other regions in Indonesia. There are four regions (4, 5, 6 and 7) located in Java Island. Regions located outside the Java Island are region 1,2,3,8,9,10,11 and12. Region 13 has a mixed combination of a province in Java and two provinces outside Java, thus we categorized it as a separate region (mixed region).

We estimate the number of predicted populations of risk of diabetes and hypertension by multiplying the regional prevalence with the projected number of populations in the region at the same year. For example, to calculate the predicted population at risk of Diabetes in region 6 (Central Java and Yogyakarta) at 2014, we multiply the prevalence of the region of 3% with projected number of populations in region 6 in 2014 (27,993,000), resulted in 839,790 persons. (See Table 1).

**Table 1:** Characteristics of BPJS regions based on population at risk of diabetes and hypertension.

BPJS Region	Provinces	Disease Prevalence*		2014					2015					2016				
		Diabetes	Hypertension	Projected Population at the year (x 1000) †	Population at risk^#^		Registered Participants	Prolanis Coverage (%)	Projected Population at the year (x 1000) †	Population at risk^#^		Registered Participants	Prolanis Coverage (%)	Projected Population at the year (x 1000) †	Population at risk^#^		Registered Participants	Prolanis Coverage (%)
					Diabetes	Hypertension				Diabetes	Hypertension				Diabetes	Hypertension		
*TOTAL*		**2.4**	**25.8**	**181,326**	**4,351,824**	**46,782,108**	**11,079**	**0.02**	**184,902**	**4,437,648**	**47,704,716**	**67,607**	**0.13**	**189,230**	**4,541,520**	**48,821,340**	**260,364**	**0.49**
*SUBTOTALS*	**JAVA**	**3.0**	**29.4**	**102,682**	**3,080,460**	**30,188,508**	**8,758**	**0.03**	**104,099**	**3,122,970**	**30,605,106**	**52,286**	**0.16**	**106,063**	**3,181,890**	**31,182,522**	**186,161**	**0.54**
*4*	Jakarta, Banten^, West Java^	3.0	29.4	28,447	853,410	8,363,418	349	< 0.01	28,931	867,930	8,505,714	2,098	0.02	29,970	899,100	8,811,180	12,195	0.13
5	West Java^	2.0	29.4	16,693	333,860	4,907,742	414	0.01	17,008	340,160	5,000,352	7,801	0.15	17,323	346,460	5,092,962	16,789	0.31
6	Central Java & Yogyakarta	3.0	26.4	27,993	839,790	7,390,152	3,810	0.05	28,321	849,630	7,476,744	31,717	0.38	28,646	859,380	7,562,544	112,178	1.33
7	East Java	2.5	26.2	29,549	738,725	7,741,838	4,185	0.05	29,838	745,950	7,817,556	10,670	0.12	30,123	753,075	7,892,226	44,999	0.52
*SUBTOTALS*	**OUTSIDE JAVA**	**3.7**	**30.8**	**65,420**	**2,420,540**	**20,149,360**	**2,311**	**0.01**	**67,334**	**2,491,358**	**20,738,872**	**15,027**	**0.06**	**68,449**	**2,532,613**	**21,082,292**	**69,052**	**0.29**
1	Aceh & North Sumatera	2.6	24.7	12,687	329,862	3,133,689	96	< 0.01	12,902	335,452	3,186,794	1,555	0.04	13,119	341,094	3,240,393	8,259	0.23
2	West Sumatera, Jambi & Riau	1.8	22.6	10,198	183,564	2,304,748	254	0.01	10,430	187,740	2,357,180	3,678	0.14	10,665	191,970	2,410,290	14,907	0.57
3	Bengkulu, Bangka Belitung & South Sumatera	2.5	30.9	7,888	197,200	2,437,392	61	< 0.01	8,061	201,525	2,490,849	779	0.03	8,209	205,225	2,536,581	4,668	0.17
8	South Kalimantan, Central Kalimantan, East Kalimantan & North Kalimantan^1^	2.7	30.8	7,406	199,962	2,281,048	10	< 0.01	7,985	215,595	2,459,380	2,125	0.08	8,186	221,022	2,521,288	10,936	0.40
9	Moluks, West Sulawesi, South Sulawesi & Southeast Sulawesi	2.8	28.1	9,551	267,428	2,683,831	895	0.03	9,725	272,300	2,732,725	2,787	0.09	9,897	277,116	2,781,057	10,790	0.35
10	Gorontalo, North Moluks, Central Sulawesi & North Sulawesi	3.7	29.0	5,316	196,692	1,541,640	58	< 0.01	5,415	200,355	1,570,350	1,313	0.07	5,512	203,944	1,598,480	10,747	0.60
11	Bali, West Nusa Tenggara & East Nusa Tenggara	3.3	24.3	9,667	319,011	2,349,081	770	0.03	10,036	331,188	2,438,748	2,263	0.08	10,006	330,198	2,431,458	7,701	0.28
12	Papua & West Papua	2.3	20.5	2,708	62,284	555,140	167	0.03	2,782	63,986	570,310	527	0.08	2,856	65,688	585,480	1,044	0.16
13	**Mixed Region** (Banten^, West Kalimantan & Lampung)^2^	**NA**	**NA**	**NA**	**NA**	**NA**	**NA**	**NA**	**13,470**	**215,520**	**3,812,010**	**294**	**0.01**	**14,718**	**235,488**	**4,165,194**	**5,151**	**0.12**

Note:

* The regional prevalence is taken from the highest disease prevalence of provinces located in the region. Data based on from Indonesian National Basic Health Survey 2013 (RISKESDAS) [5]

^†^ Projected population at the year based on the Indonesian population projection 2010–2035 released by Indonesian Central Statistics Agency[6];

^#^ Calculated by multiplying the number of projected population at the year with disease prevalence;

^ we included half of total predicted population of Banten region 4, and another half in region 13. We also included half of total predicted population of West Java in region 4, and another half in region 5.

^1^ At 2013 this province has not yet been established^;^

^2^ Region is established in 2015.

We took the regional prevalence values from the highest disease prevalence of provinces located in specific BPJS region (based on data from Indonesian National Basic Health Survey 2013 (RISKESDAS) [5]); while the projected number of population in the region is the sum of projected population of the provinces included in the specific BPJS region (based on the Indonesian population projection 2010–2035 released by Indonesian Central Statistics Agency [6]). As there are two provinces, namely Banten and West Java that were split by BPJS within two different regions; we included half of total predicted population of Banten region 4, and another half in region 13. Subsequently, we included half of total predicted population of West Java in region 4, and another half in region 5.

RISKESDAS is a nationwide health survey that is conducted every 5–6 years by the Ministry of Health of Indonesia since 2007. The 2013 RISKESDAS was a household survey that covers 33 provinces of Indonesia. The report was recently updated in 2018 (at the time of this article is written, complete official report is not yet released). The survey described the situation at the national and provincial levels, and also at the district / city level. In the RISKESDAS, history of diabetes mellitus was asked to subjects aged 15 years and over. Prevalence of diabetes mellitus are presented in two ways: first, based on respondents’ acknowledgment; second based on the results of interviews in the form of prevalence based on cases of diseases diagnosed by doctors / health workers. [5] In this study, we took the latter.

Hypertension was assessed through 2 methods, interview and blood pressure measurement. We took the hypertension prevalence per province based on the latter. Blood pressure measurements are performed using a standardized digital measuring device by trained enumerators. Each respondent is measured at least 2 times. If the results of the second measurement differ from ≥10 mmHg compared to the first measurement, then the third measurement is carried out. The two-measurement data with the smallest difference with the last measurement are calculated as a mean as a measure of tension. [5]

For the projected number of population at the year 2014 to 2016, we used the Indonesian population projection 2010–2035 released by Central Statistics Agency.[6] The projection was made base on the results of the 2010 Indonesian Population Census. This projection is made based on assumptions about birth trends, death, and migration inter-provincial populations are most likely to occur over the next 25 years. First, the projected population of Indonesia were calculated, then projection was made per province. The projection results are discussed in the technical team formed by Central Statistics Agency, and then discussed in a team meeting consisting of officials from National agencies, academics and other relevant agencies.

To see whether the program covers a substantial part of those who already at risk, we calculated the percentage of coverage by dividing the number of registered participants with the sum predicted populations at risk for diabetes and hypertension at the same year. For example, to calculate the percentage of coverage in region 6 (Central Java and Yogyakarta) at 2014, we divide the registered participants (3,810) with the sum of predicted populations at risk for diabetes (839,790) and the predicted populations at risk for hypertension (7,390,152) in the region at 2014; resulted in 0.05% of coverage (see Table 1).

The cost incurred from Prolanis include the cost for screening for health status monitoring (for diabetes and hypertension), activity club and health education. The costs for home visit was already included under the capitation fund for primary healthcare centers, hence was excluded in this study. Health status monitoring included examination for random and fasting blood glucose test once per month, HbA1c test every 6 months, and blood chemistry test every year. Monitoring for hypertension was conducted regularly in each health status monitoring visits and activity club, thus was not expressed in costs incurred by BPJS. Health education/medical counselling was provided every month by general physicians at primary health care center. Physical-activity clubs are group physical activities conducted regularly by the primary health care centers for the Prolanis participants. In the physical-activity club, cost may incur from sports coach fees and operational costs. We categorized the costs by regions and locations (Java/Outside Java and Mixed regions) and calculate the cost per capita for each activity by year. The cost per capita was calculated by dividing the cost of the activity by the number of participants in each activity. All costs were in Indonesian rupiahs.

Results were presented in tables and analyze descriptively.

## Results

Characteristics of regions based on population at risk of diabetes and hypertension are described in Table 1. In general, data from BPJS showed an increase trend number of participants from around 11,000 in 2014 to more than 250,000 in 2016. About 70% of participants were living in Java island.

The national prevalence of diabetes diagnosed by medical doctors in 2013 was 2.4%, while the national prevalence of hypertension was 25.8%.[5] Majority of provinces located outside Java had diabetes and hypertension prevalence lower than national prevalence.

In 2014, all over, the Prolanis covers 0.02% population at risk. In 2015 and 2016, although still very low, the proportion of national coverage was increased to 0.13% and 0.49%. In general, every year, regions in Java always had a higher coverage than regions outside Java (2014: 0.03% vs. 0.01%; 2015: 0.16% vs. 0.06%; 2016: 0.54% vs.0.29%). (Table 1).

From Table 1, we can see that in 2014, regions 6 and 7 had the highest percentage of coverage (both 0.05%). In 2015, region 2 (0.14%), 5 (0.15%), and 6 (0.38%) had the highest percentage of coverage and had a higher percentage coverage than the national coverage. In 2016, region 2 (0.57%), 6, (1.33%) and 7 (0.52%) had the highest percentage of coverage. Except for region 2, all the mentioned above are located in Java.

In Table 2, we presented the utilization of Prolanis activities per region by year. The number of each activities are rising from year 2014 to 2015, however, since the number of Prolanis members are also increasing greatly, the utilization rate of all activities seems lower in 2016 compared to 2015. The utilization rate for activity club is always higher compared to other activities in all year for all regions.

**Table 2:** Utilization of Prolanis activities per region by year.

BPJS Region	2014							2015							2016						
	Registered Participants	Health Status Monitoring		Activity Club		Education Session		Registered Participants	Health Status Monitoring		Activity Club		Education Session		Registered Participants	Health Status Monitoring		Activity Club		Education Session	
		N	Utilization Rate	N	Utilization Rate	N	Utilization Rate		N	Utilization Rate	N	Utilization Rate	N	Utilization Rate		N	Utilization Rate	N	Utilization Rate	N	Utilization Rate
*TOTAL*	**11,079**	**9,235**	**2.1**	**33,580**	**10.4**	**19,942**	**6.2**	**67,607**	**6,766**	**5.4**	**66,100**	**20.7**	**42,283**	**2.9**	**260,364**	**235,524**	**1.3**	**104,605**	**1.4**	**47,264**	**0.5**
**JAVA**	**8,758**	**5,995**	**0.7**	**12,822**	**1.5**	**7,773**	**0.9**	**52,286**	**4,177**	**0.1**	**19,839**	**0.4**	**27,139**	**0.5**	**186,161**	**234,656**	**1.3**	**39,636**	**0.2**	**23,239**	**0.1**
4	349	NA	NA	1,313	3.8	711	2.0	2,098	132	0.1	3,202	1.5	1,958	0.9	12,195	NA	**0.0**	3,242	**0.3**	1,743	0.1
5	414	5,995	14.5	2,132	5.1	1,162	2.8	7,801	NA	NA	3,011	0.4	1,875	0.2	16,789	NA	**0.0**	9,164	**0.5**	2,958	0.2
6	3,810	NA	NA	2,470	0.6	4,410	1.2	31,717	4,045	0.1	8,598	0.3	16,929	0.5	112,178	NA	**0.0**	16,994	**0.2**	11,745	0.1
7	4,185	NA	NA	6,907	1.7	1,490	0.4	10,670	NA	NA	5,028	0.5	6,377	0.6	44,999	234,656	**5.2**	10,236	**0.2**	6,793	0.2
**OUTSIDE JAVA**	**2,311**	**3,240**	**1.4**	**20,758**	**9.0**	**12,169**	**5.3**	**15,027**	**1,040**	**0.1**	**41,080**	**2.7**	**14,722**	**1.0**	**69,052**	**868**	**0.0**	**63,631**	**0.9**	**23,757**	0.3
1	96	1,920	20.0	4,607	48.0	4,180	43.5	1,555	NA	NA	8,882	5.7	2,778	**1.8**	8,259	638	**0.1**	9,690	**1.2**	3,128	0.4
2	254	458	1.8	2,434	9.6	422	1.7	3,678	393	0.1	4,529	1.2	1,083	**0.3**	14,907	148	**0.0**	7,033	**0.5**	1,722	0.1
3	61	NA	NA	2,119	34.7	797	13.1	779	NA	NA	2,947	3.8	654	**0.8**	4,668	NA	**0.0**	5,913	**1.3**	1,322	0.3
`8	10	778	77.8	2,374	237.4	1,670	167.0	2,125	NA	NA	6,976	3.3	2,758	**1.3**	10,936	NA	**0.0**	11,725	**1.1**	3,635	0.3
9	895	NA	NA	2,351	2.6	1,910	2.1	2,787	NA	NA	6,412	2.3	3,399	**1.2**	10,790	NA	**0.0**	11,134	**1.0**	4,799	0.4
10	58	84	1.4	1,359	23.4	1,306	22.5	1,313	647	0.5	4,780	3.6	1,835	**1.4**	10,747	44	**0.0**	8,314	**0.8**	5,631	0.5
11	770	NA	NA	5,408	7.0	1,831	2.4	2,263	NA	NA	5,530	2.4	1,750	**0.8**	7,701	38	**0.0**	8,222	**1.1**	2,933	0.4
12	167	NA	NA	106	0.6	53	0.3	527	NA	NA	1,024	1.9	465	**0.9**	1,044	NA	**0.0**	1,600	**1.5**	587	0.6
**Mixed**	**10**	NA	NA	NA	NA	NA	NA	**294**	**1,549**	**5.3**	**5,181**	**17.6**	**422**	**1.4**	**5,151**	NA	**0.0**	**1,338**	**0.3**	**268**	0.1
13	10	NA	NA	NA	NA	NA	NA	294	1,549	5.3	5,181	17.6	422	**1.4**	5,151	NA	**0.0**	1,338	**0.3**	268	0.1

Note: NA = Data is not available

In Table 3, we showed the total expenditure for Prolanis program had increased from 29,8 trillion IDR (2,1 million USD) in 2014 to 83,5 trillion IDR (5,8 million USD) in 2016. This was in line with the increase number of Prolanis participants more than twenty times from year 2014 (11,079 persons) to 2016 (260,364 persons). However, if we calculated the individual cost, the cost per capita was actually decreased from 475,033 to 215,089.5 IDR per capita (54.7% decrease).

**Table 3:** Total costs (in Indonesian Rupiah) of Prolanis program in 2014–2016.

	2014		2015		2016	
	Total	per capita	Total	per capita	Total	per capita
**Cost for Health Status Monitoring**	153,312,000	16,601	67,564,000	9,986	1,213,223,500	5,151
**Regions in Java**	103,655,000	17,290	24,752,000	5,926	1,195,526,000	5,095
**Regions outside Java**	49,657,000	15,326	15,237,000	14,651	17,697,500	20,389
**Mixed Region**	NA	NA	27,575,000	17,802	NA	NA
**Cost for activity club**	14,672,060,673	436,929	29,113,659,166	440,449	47,272,407,525	451,913
**Regions in Java**	3,899,891,395	304,156	9,188,577,521	463,157	17,211,845,645	434,248
**Regions outside Java**	10,772,169,278	518,941	19,033,653,895	463,331	27,207,916,376	427,589
**Mixed Region**	NA	NA	891,427,750	172,057	2,852,645,504	2,132,022
**Cost for education Session**	14,986,291,695	751,494	31,797,621,590	752,019	35,053,617,557	726,289
**Regions in Java**	6,902,215,784	887,973	15,989,780,465	589,181	15,518,705,239	640,237
**Regions outside Java**	8,084,075,911	664,317	15,301,988,769	1,039,396	19,009,408,768	800,160
**Mixed Region**	NA	NA	505,852,356	1,198,702	525,503,550	1,960,834
**Annual total costs**	29,811,665,368	475,033	60,978,844,756	529,564.7	83,539,248,582	215,089.5

1 Indonesian Rupiah = 0.000070 USD; NA = Data is not available

## Discussion

In this study we found an increasing number of participants and activities of Prolanis within the year 2014 to 2016. Activity clubs’ activities were favorited, especially for regions located outside Java. We also observed almost triple increased of total expenditure for Prolanis program in 2016 from the annual cost in 2014 but the individual cost spent per patient has decrease for more than 50%.

The increase in the number of Prolanis participants to more than 20 folds within the year of 2014 to 2016, can be translated as something fascinating, but expected. Even after a year of implementation, the coverage of Prolanis has reached all its predicted potential population, especially in Java. Since its introduction in 2014, the popularity of Prolanis and BPJS are rising because it was promoted as free-health care service by the Indonesian government. This might cause the massive hike of utilization and claims of any service release by BPJS right after its first year of implementation. However, we can see that after 2 years of implementation, the acceptance rate was still very low for regions located outside Java island compare to regions located in Java. This might be related with the lower rate of PHCs availability outside Java. Primary health clinics are the main driver of community participations in Indonesia. In 2015, the Indonesian Ministry of Health introduced the concept of “Healthy Community Movement (*Gerakan Masyarakat Sehat* -GERMAS) that focus more heavily on non-communicable disease prevention.

The number of Prolanis claims were increase greatly for activity clubs, especially from 2014 to 2015 (33,580 vs. 66,100). This increase number of activity clubs was especially shown for regions outside Java. We did not find the right reason for this finding, expect that it the cost for activity clubs outside Java might be much cheaper than regions in Java, thus making it interesting and easier to implement by PHC as part of implementation of Prolanis in their area.

We also observed the high increase of total expenditure for Prolanis program and decrease individual cost. This might have been caused by the increase number of participants. According to BPJS expenditure report, the allocated budget for Prolanis program per total national health budget is decreasing every year from 0.54% (2014) to 0.13% (2016). This was in contrary to the cost for chronic diseases, which was the biggest contributor the total national health expenditure. It shows how concern on disease treatment grabs more attention than investing in promotive and prevention program. This certainly gives more reasons for MOH and BPJS to increase the budget of promotive and preventive programs for non-communicable disease prevention, such as Prolanis, in Indonesia.

Previous small study show the effectivity of Prolanis in controlling the progression of hypertension[7]. Our analysis had not look at clinical outcome of these patients and we acknowledged this as one of this study weakness. In order to evaluate the clinical impact of this program, more studies that look at the association of the program to clinical outcome of chronic diseases may need to be conducted. However, this was a first study who look at national data claim of Prolanis and includes the cost of the program. Conducting this research could also be beneficial for the payers as they can evaluate and monitor the effectiveness of their program. Other weakness of this study is we relied mainly on secondary data from national health security agency; while reliability on data collection remains questionable. Moreover, the claim data we have obtained from BPJS did not have more detail component of costs and some components of regions data are missing; for example, the health status monitoring claims for in 2015 and 2016. This might have been cause by underreporting by the regions. Nonetheless, we did not believe this under reporting was selective, thus is potential for selection bias. Furthermore, as we cannot get individual patients claim data due to confidentiality issues, we could not identify whether the population, within years are overlapping. Despite study weaknesses, this study provides evidence that can help implementation and expansion of similar chronic disease management, in low-middle income countries.

As conclusion, within two years of UHC implementation, there were increase covered participants and total costs, but cost per individual was decreased and there was significant difference in of cost between Java and outside Java. Further study and routine monitoring-evaluation process by health authority are needed to assess whether the cost difference would affect the service quality.
